# The Influence of Particle Size Distribution and Shell Imperfections on the Plasmon Resonance of Au and Ag Nanoshells

**DOI:** 10.1007/s11468-016-0345-8

**Published:** 2016-08-08

**Authors:** Daniel Mann, Daniel Nascimento-Duplat, Helmut Keul, Martin Möller, Marcel Verheijen, Man Xu, H. Paul Urbach, Aurèle J. L. Adam, Pascal Buskens

**Affiliations:** 1 DWI - Leibniz Institute for Interactive Materials e.V., Forckenbeckstr. 50, 52056 Aachen, Germany; 2 Department of Imaging Physics, Delft University of Technology, Lorentzweg 1, 2628 CJ Delft, The Netherlands; 3 Philips Innovation Labs, High Tech Campus 11, 5656 AE Eindhoven, The Netherlands; 4 Department of Applied Physics, Eindhoven University of Technology, P. O. Box 513, 5600 MB Eindhoven, The Netherlands; 5 The Netherlands Organisation for Applied Scientific Research (TNO), De Rondom 1, 5612 AP Eindhoven, The Netherlands

**Keywords:** Plating, Core-shell nanoparticle, Optical simulation, Mie theory, Finite element method

## Abstract

Au and Ag nanoshells are of interest for a wide range of applications. The plasmon resonance of such nanoshells is the property of interest and can be tuned in a broad spectral regime, ranging from the ultraviolet to the mid-infrared. To date, a large number of manuscripts have been published on the optics of such nanoshells. Few of these, however, address the effect of particle size distribution and metal shell imperfections on the plasmon resonance. Both are inherent to the chemical synthesis of metal nanoshells and therefore to a large extent unavoidable. It is of vital importance to understand their effect on the plasmon resonance, since this determines the scope and limitations of the technology and may have a direct impact on the application of such particles. Here, we elucidate the effect of particle size distribution and imperfections in the metal shell on the plasmon resonance of Au and Ag nanoshells. The size of the polystyrene core and the thickness of the Au and Ag shells are systematically varied to study their influence on the plasmon resonance, and the results are compared to values obtained through optical simulations using extended Mie theory and finite element method. Discrepancies between theory and practice are studied in detail and discussed extensively. Quantitative information on the minimum thickness of the metal shell, which is required to realize a satisfactory plasmon resonance of a metal nanoshell, is provided for Au and Ag.

**Electronic supplementary material:**

The online version of this article (doi:10.1007/s11468-016-0345-8) contains supplementary material, which is available to authorized users.

## Introduction

Sub-micron-sized metal particles are of interest for a broad variety of applications ranging from chemical and biological sensing [1–3] to medical applications [4–6], photocatalysis [7–10], surface-enhanced Raman spectroscopy [10–12], sunlight harvesting [13–15], and light trapping [15–18]. For all of these applications, their localized plasmon resonance is the property of interest, and the particles are used for plasmonic absorption and/or scattering. For every application, the plasmon resonance of the metal nanoparticle needs to be tailored to match the emission of the light source, enabling an efficient energy transfer to the metal nanoparticle. The light source may be monochromatic or polychromatic and emit ultraviolet (UV), visible (VIS), and/or infrared (IR) light. In addition to the wavelength of maximum extinction, the peak width of the plasmon resonance is of importance, e.g., in cases in which the energy of a specific and narrow wavelength window should be selectively transferred to the metal particle. The only class of metal-containing nanoparticles that allows tuning of the resonance wavelength in a broad spectral regime, ranging from the UV into the mid-IR, is spherical particles with a dielectric core and a metal shell—so-called metal nanoshells [19, 20]. For such particles, the plasmon resonance is dependent on the dielectric constant of the core [21] and shell material [22, 23], the diameter of the core, the thickness of the metal shell, and the refractive index of the surrounding medium [24–26].

**Fig. 1:**
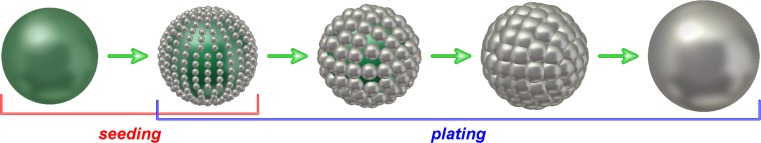
Schematic representation of the formation of metal nanoshells (*green* = polystyrene, *silver* = metal)

To date, a large number of manuscripts have been published on the optics of metal nanoshells. The most common core materials used are silica [27–34] or polystyrene [35–42] with metal shells made of Au [27–29, 35–39], Ag [30–33, 39–43], or Cu [34]. Yong et al. [39] reported a mainly qualitative analysis of the shell variation for silver or gold shells deposited onto gold-decorated polystyrene nanospheres. The measured optical response of the synthesized nanoparticles presents a broader response when compared to the theoretical result obtained by extended Mie’s theory. None of these studies, however, perform a systematic study of the effect of particle size distribution and metal shell imperfections on their plasmon resonance. Both are inherent to the chemical synthesis of metal nanoshells and therefore to a large extent unavoidable. It is of vital importance to understand their effect on the plasmon resonance, since this determines the scope and limitations of the technology and may have a direct impact on the application of such particles. Typical synthesis routes involve the synthesis of dielectric core particles, followed by deposition of metal on the surface of these particles through reduction of corresponding metal salts. For the resulting metal nanoshells, the cores are not uniform in size, and the metal shell is incomplete and rough. Latter is caused by the seed-and-growth approach applied for forming such metal shells, in which the surface of the dielectric core particle is first decorated with individual metal seeds, which subsequently grow and form a metal shell through coalescence of metal islands (Fig. 1). To date, it is not reported in literature which minimum shell thickness is required to obtain a metal shell that is sufficiently complete to realize the plasmon resonance of a metal nanoshell. The required minimum shell thickness is likely to be dependent on the type of metal.

Here, we report the effect of particle size distribution and metal shell imperfections of Au and Ag nanoshells on their localized plasmon resonance. We focus on the effect of non-uniformity in size of the core particles and aim at elucidating the metal layer thickness required to realize the optical properties of metal nanoshells. We selected Au and Ag nanoshells, which are mostly applied, as study systems.

We present the design and synthesis of glucose-functionalized polystyrene particles tailored for the selective deposition of Ag and Au through reduction of corresponding metal salts. The Au and Ag shells, which comprise the same polystyrene core, are realized in a two-step seed growth procedure. Using scanning electron microscopy (SEM), we analyze the degree of surface coverage of the polystyrene particles with Ag or Au grains for various thicknesses of metal shells. The sizes of the polystyrene core and the Au and Ag shells are systematically varied to study their influence on the plasmon resonance, and the results are compared to values obtained through optical simulations using extended Mie theory and finite element method. Discrepancies between theory and practice are studied in detail and discussed extensively. Quantitative information on the minimum thickness of the metal shell, which is required to realize the optics of a metal nanoshell, is provided for Au and Ag.

## Results and Discussion

### Metal Nanoshells: Synthesis and Structural and Compositional Analysis

In 2014, our group reported the synthesis of glucose-functionalized polystyrene particles, tailored for the decoration with Ag nanoparticles [44]. Such polystyrene particles were used as dielectric cores for the synthesis of Au and Ag nanoshells (Scheme S1 and Table S1).

### Ag Nanoshells

#### Ag Seeding

**Fig. 2:**
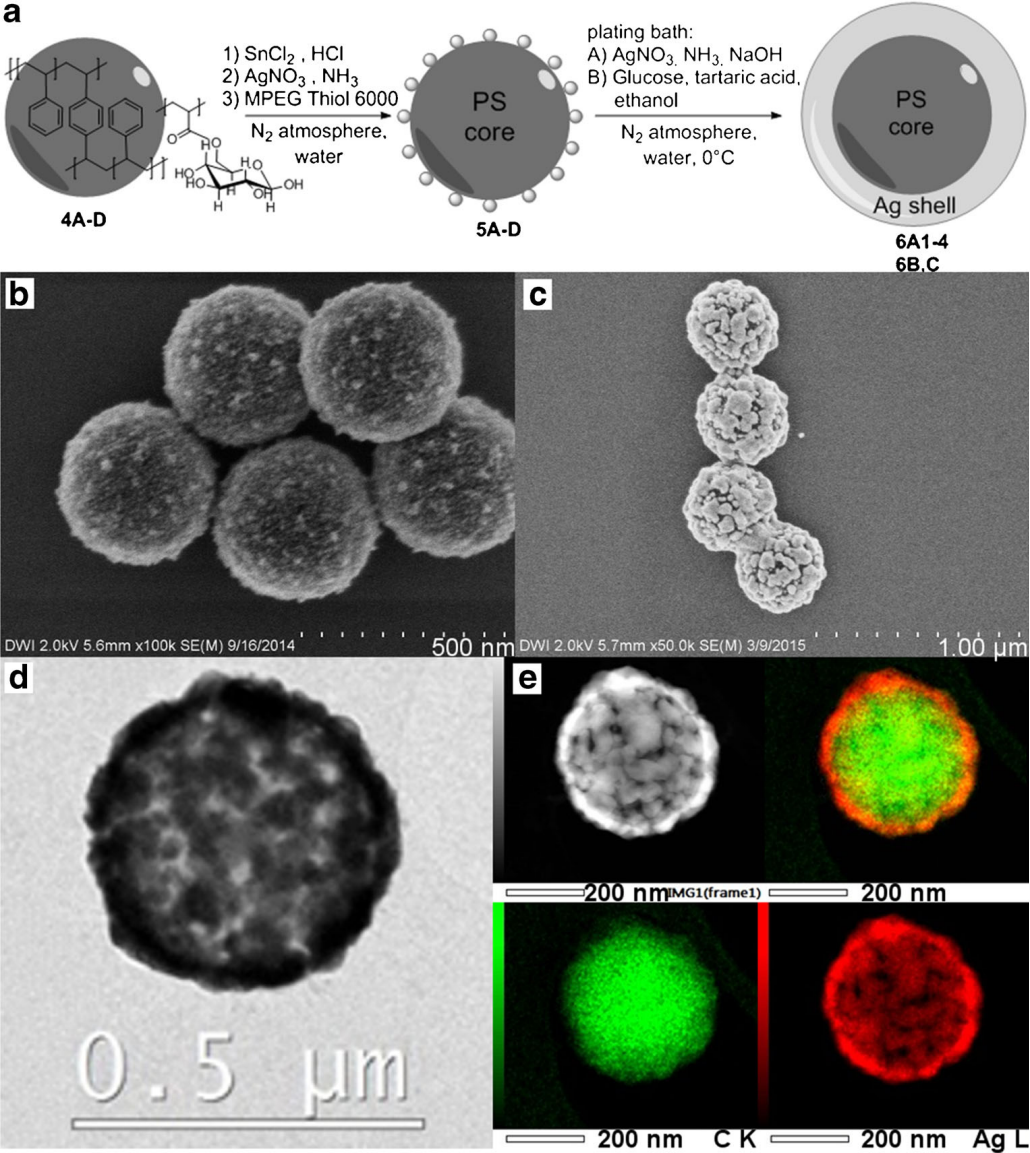
**a** Schematic representation of Ag seeding and plating on polystyrene nanoparticles. FESEM images of polystyrene-Ag composite particles **b** after seeding (5A) and **c** after subsequent electroless plating (6A1), plus **d** HR-TEM and **e** STEM-HAADF image and ED

Polystyrene particles dispersed in water were treated with tin(II) chloride. The Sn^2+^ ions coordinated to the particle surface, which was demonstrated using zeta potential measurements [44]. They served as nucleation points for the reductive deposition of Ag. After centrifugation and washing to remove the excess of Sn^2+^ ions in solution, the aqueous dispersion of tin-modified polystyrene particles was added to an aqueous silver(I) diammine solution. Through reduction of Ag ions by the glucose groups on the polystyrene particle surface, small, homogeneously distributed Ag seeds were formed instantly (5A, Fig. 2). Poly(ethylene glycol) methyl ether thiol *M*_n_ 6000 (MPEG Thiol 6000) was added to the reaction mixture, and the polystyrene-Ag composite particles were purified through centrifugation and washing. The Ag seeding and subsequent plating were performed under inert gas atmosphere to prevent oxidation of Ag, since (partial) oxidation has a strong influence on the plasmon resonance [45].

#### Ag Plating

The polystyrene particles decorated with Ag nanoseeds (5A) were subjected to an electroless Ag plating bath, in which glucose was used to reduce silver(I) diammine complex directly onto the Ag seeded polystyrene particles. Through growing of the Ag seeds in the plating bath and ultimate coalescence of the growing Ag islands, a Ag shell was formed. Ag nanoshell 6A1 with an average core diameter of 391 nm and shell thickness of 19 nm was synthesized (Fig. 2c–e and Table 1). The reaction was carried out under N_2_ atmosphere to prevent oxidation of the Ag shell. To prevent agglomeration, the reaction mixture was diluted to a polystyrene concentration of 0.014 mg/mL, and after the reaction, MPEG Thiol 6000 was added as stabilizer.

**Table 1:** Dimensions of the metal nanoshells

Particle	Metal	Core diameter^a^ [nm]	Shell thickness^b^ [nm]	Metal grain size^c^ [nm]	Surface coverage^d^ [%]
6A1	Ag	391	19	82	91.3
6A2	Ag	391	31	84	94.3
6A3	Ag	391	38	96	95.6
6A4	Ag	391	45	136	97.7
6B	Ag	339	35	87	94.4
6C	Ag	218	30	60	94.1
8A1	Au	391	11	41	98.9
8A2	Au	391	14	44	98.0
8A3	Au	391	20	39	96.7
8A4	Au	391	24	42	99.2
8B	Au	339	14	35	98.0
8C	Au	267	15	38	98.5
8D	Au	218	15	35	98.3

^a^Average core diameter was calculated from FESEM images of polystyrene particles, measuring at least 200 particles

^b^Average shell thickness was calculated by subtraction of the average core radius from the average radius of the nanoshells. Latter was calculated from FESEM images of Ag and Au nanoshells, measuring at least 200 particles

^c^Metal grain size is defined as the average diameter of metal islands on the polystyrene surface, assuming those to be discoidal particles, and was calculated from FESEM images measuring metal grains on the surface of at least 200 particles

^d^Surface coverage was calculated from FESEM images measuring at least 200 particles

The Ag nanoshells were stable during centrifugation up to 6000 rpm and treatment with an ultrasonic bath. They segregated because of gravity but were fully re-dispersible for at least 4 weeks after synthesis.

#### Structural Analysis of Nanoshell 6A1

The selective deposition of Ag on the polystyrene particle surface was demonstrated using field emission scanning electron microscopy (FESEM) measurements. Using FESEM analyses, we demonstrated that all latex particles were covered with metal (Fig. 2b, c). We furthermore showed, by conducting dynamic light scattering (DLS) measurements and analyzing the particle diameter from FESEM images, that the nanoshells have a narrow size distribution of <0.05, similar to the size distribution of the polystyrene core particles (Table S2). To obtain more detailed information on the Ag shell of particle 6A1, we performed high-resolution transmission electron microscopy (HR-TEM) and scanning transmission electron microscopy-high angle annular dark field (STEM-HAADF) imaging, combined with energy-dispersive X-ray spectroscopy (EDS) measurements (Fig. 2d, e). Performing EDS measurements, we confirmed that the shell consists, as expected, of Ag. Analyzing TEM measurements, we showed that the seed particles grew during the electroless plating and ultimately coalesced to form a cohesive shell. Small holes, however, were still visible in the Ag shell. By analyzing the surface structure of nanoshell 6A1 using the FESEM images, we calculated an average coverage of the polystyrene particle surface of 91.3 % and an average Ag grain size of 82 nm (Table 1). The island-like growth of the Ag seeds, with an average size of <20 nm from particles 5A with a surface coverage of <50 %, to form a metal shell is still visible after coalescence in form of the cauliflower-type surface structure. To demonstrate that we prevented oxidation of the Ag surface through working under nitrogen atmosphere, we performed X-ray photoelectron spectroscopy (XPS) on nanoshells synthesized in air and under inert gas atmosphere. The XPS spectrum for the nanoshells synthesized under inert gas atmosphere showed for the Ag 3d_5/2_ and Ag 3d_3/2_ core-level peaks each a single narrow peak (Fig. S1). The Ag 3d_5/2_ peak could be reasonably fitted at 368.26 eV with a full width of half maximum (FWHM) of 0.70 eV, which is in agreement with the reference value for Ag^0^ [46]. For the nanoshells synthesized in air, the two Ag 3d peaks in the XPS spectrum were much broader with a small shoulder each. The Ag 3d_5/2_ peak could be fitted with two peaks, one centered at 368.25 (FWHM = 0.90 eV) for Ag^0^ and the other centered at 367.52 eV (FWHM = 0.84). This position is in good agreement with reference values for Ag^I^ in Ag_2_O [46]. By comparing the optical properties of Ag nanoshells synthesized under N_2_ atmosphere to those synthesized in air, we showed that oxidation has indeed a strong influence on the plasmon resonance (Fig. S2).

#### Increase in Shell Thickness

**Fig. 3:**
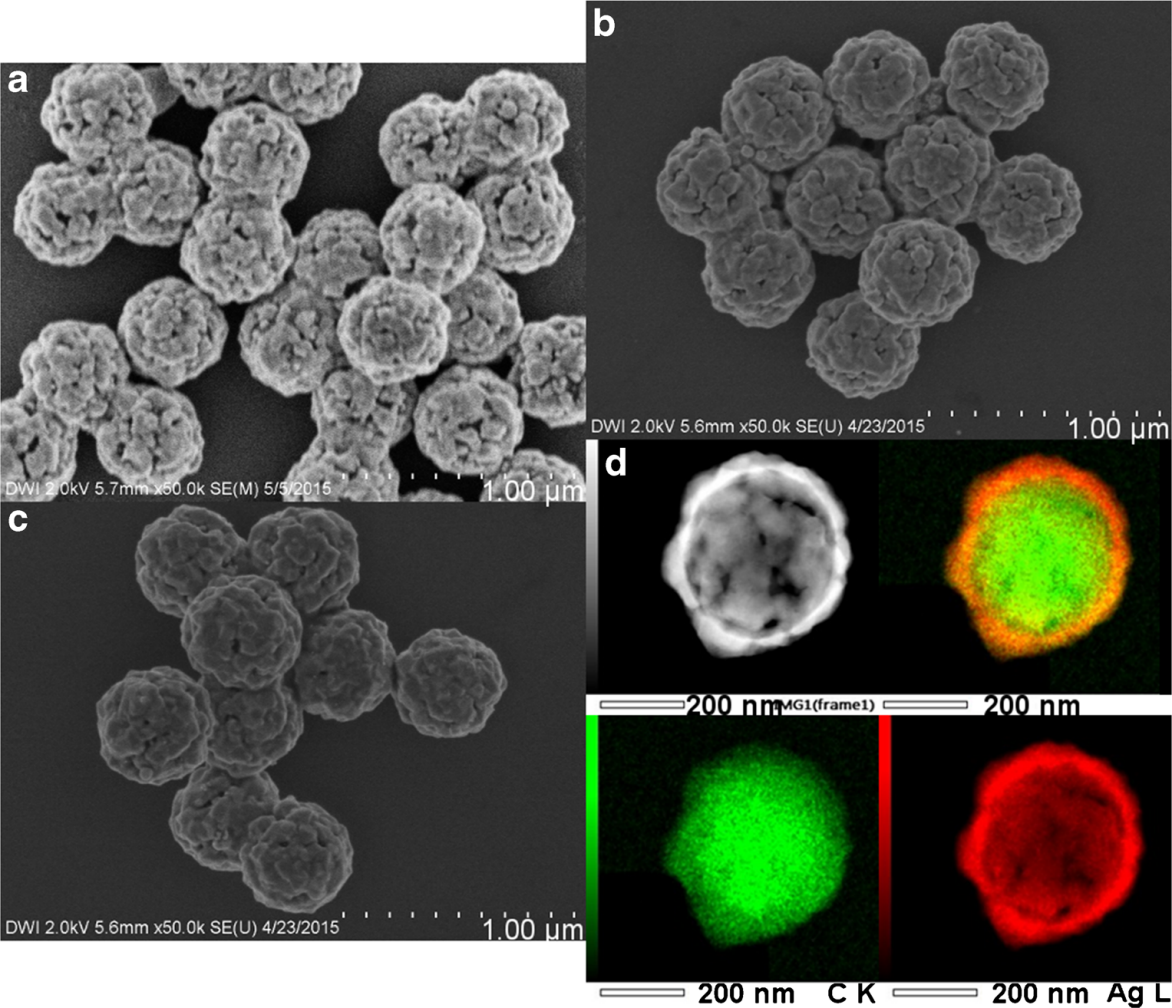
FESEM images of Ag nanoshells with same core size and different shell thickness **a** 6A2 with 31 nm, **b** 6A3 with 38 nm, and **c** 6A4 with 45 nm shell thickness, plus **d** STEM-EDX images of nanoshells 6A4

To increase the shell thickness of the polystyrene-Ag particles, we changed the amount of silver(I) diammine and glucose solution in the plating bath. We showed that an increase in silver(I) diammine used in the plating bath led to an increase in shell thickness (Table S3). By increasing the amount of silver(I) diammine while keeping the ratio Ag/glucose constant, we synthesized nanoshells with a Ag shell thickness up to 45 nm (6A2–4, Fig. 3 and Table 1). MPEG Thiol 6000 was added for stabilization of the Ag nanoshells in aqueous dispersion. All Ag nanoshells showed similar dispersion stability (Table S2). With FESEM, HR-TEM, STEM-HAADF, and EDS measurements, we showed that an increase in shell thickness led to a shell with less holes and a higher degree of surface coverage (Fig. 3). This was confirmed by surface structure analysis using the electron microscopy images. Furthermore, we demonstrated that an increase in shell thickness also led to an increase in grain size (Table 1).

#### Decrease in Core Size

**Fig. 4:**
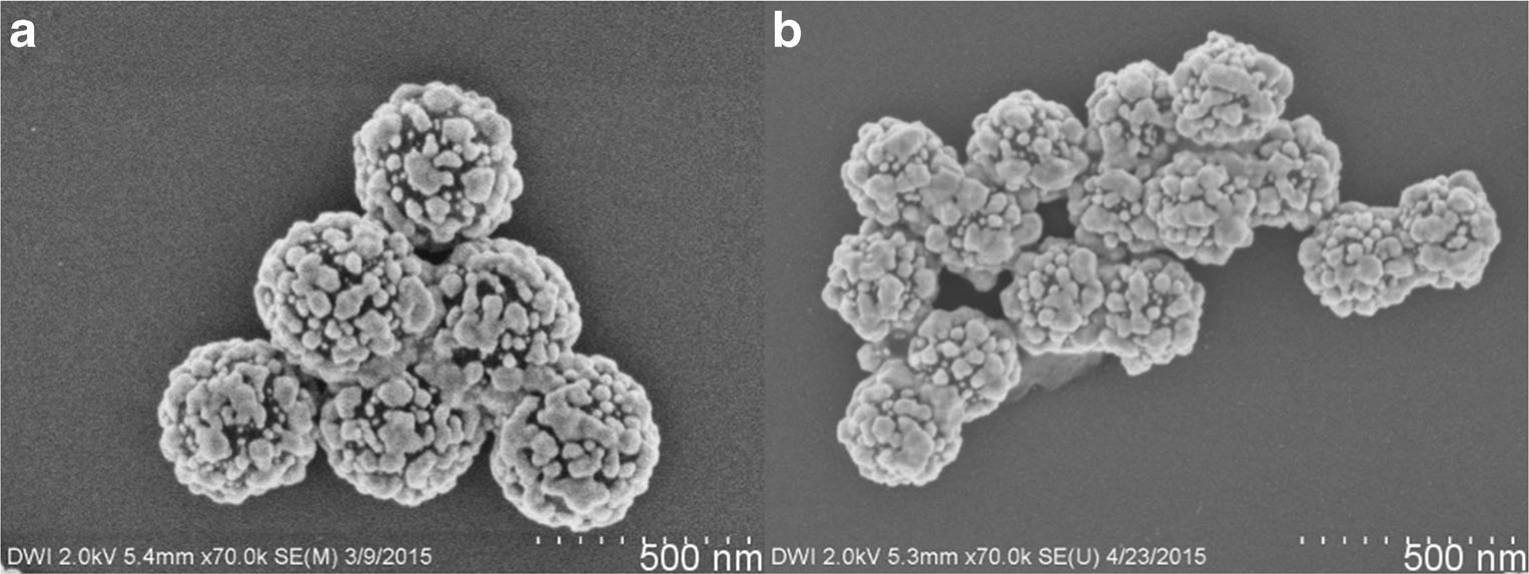
FESEM images of Ag nanoshells with different core sizes and same shell thickness **a** 6B with 339 nm and **b** 6C with 218 nm core diameter

We already showed in a previous publication that the Ag seeding method can be used on polystyrene particles with various core sizes [44]. Performing the above-described reactions using particles with average diameter of 218 nm (4D) and 339 nm (4B), we synthesized Ag nanoshells with different core diameters and similar shell thickness of 30–35 nm (6B, C, Fig. 4 and Table 1). To realize similar shell thickness, the concentration of the latex particles was adjusted so that for all experiments the same total polystyrene particle surface area was used (Tables S1 and S3). Ag was selectively deposited on all core particles, and after addition of MPEG Thiol 6000, all nanoshells showed similar stability to centrifugation and ultrasonic treatment. Analyzing the particle size using FESEM images and DLS measurements, no signs of agglomeration were detected (Table S2). When decreasing the core size from 339 to 218 nm at similar shell thickness between 30 and 35 nm, the resulting Ag nanoshells increasingly deviate from a perfect spherical morphology (Fig. 4).

### Au Nanoshells

#### Au Seeding

**Fig. 5:**
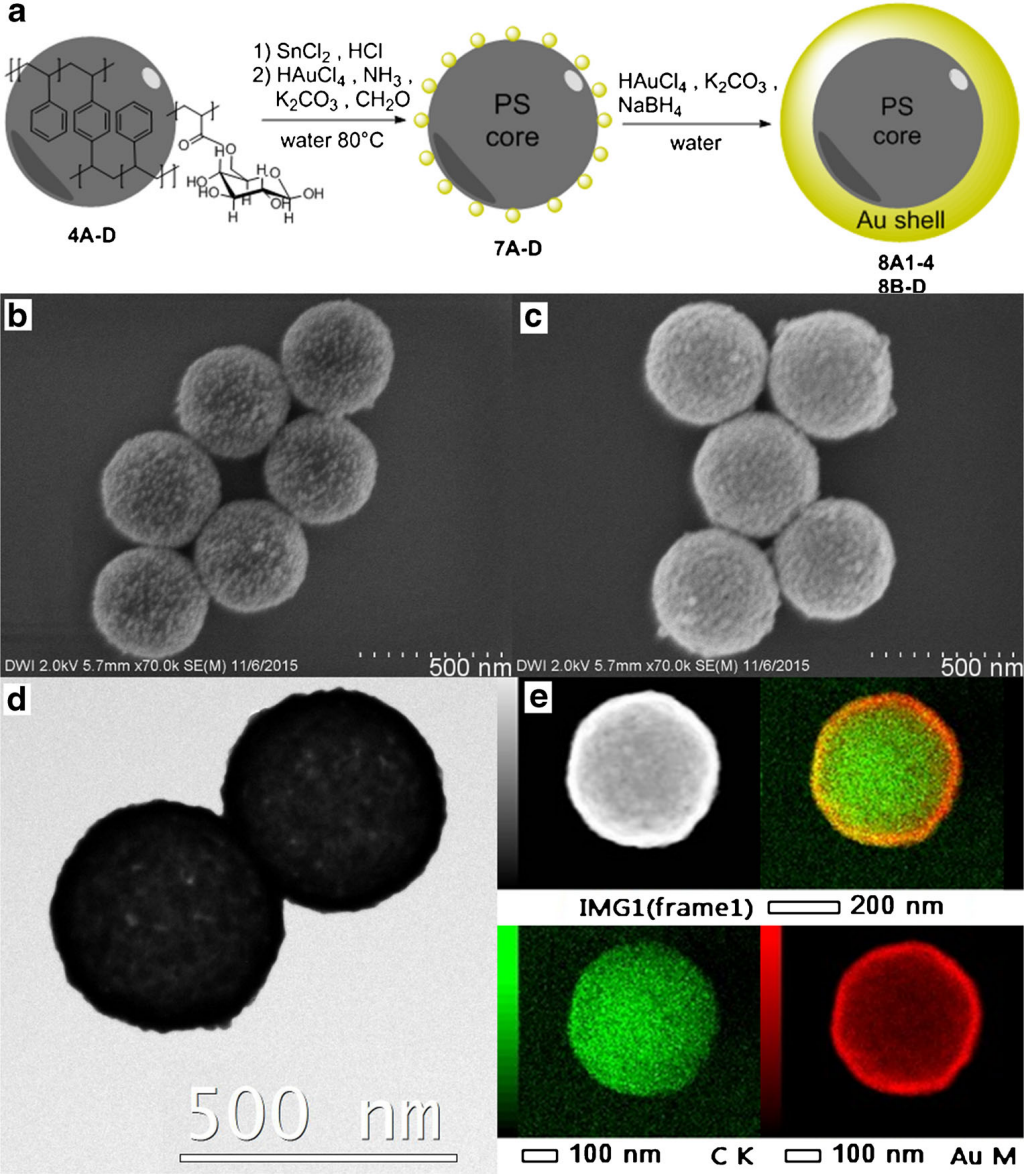
**a** Schematic representation of Au seeding and plating on polystyrene nanoparticles. FESEM images of polystyrene-Au composite particles **b** after seeding (7A) and **c** after subsequent electroless plating (8A1), plus **d** HR-TEM and **e** STEM-HAADF image and EDS elemental maps of nanoshells 8A1

For the synthesis of Au nanoshells, we used chloroauric acid as metal salt. The synthetic route consists of a Au seeding step with subsequent electroless gold plating (Fig. 5a). In the seeding step, the polystyrene particles were treated with tin(II) chloride [44]. After removing excess of tin ions by centrifugation, the pH of the solution was adjusted to 6 through addition of 0.1 M aqueous ammonia. The dispersion of tin-modified polystyrene particles was subsequently added to an aqueous solution of chloroauric acid and potassium carbonate. After adding a freshly prepared 0.7 M formaldehyde solution and stirring for 1 h at 80°C, polystyrene particles with small, evenly distributed Au seeds on the surface (7A) were synthesized (Fig. 5b).

#### Au Plating

The polystyrene-Au composite particles 7A, obtained after seeding, were added to an electroless Au plating bath. The plating bath was prepared through overnight ageing of an aqueous 0.4 M chloroauric acid solution with potassium carbonate to form a gold(III) hydroxide solution [27]. After combining the gold(III) hydroxide solution with the just synthesized Au-seeded polystyrene particles, a 7 M sodium borohydride solution was added during 1 h with a syringe pump. Under these conditions, the Au seeds attached to the polystyrene particle surface served as nucleation points for further Au deposition resulting in the formation of a cohesive Au shell around the polystyrene particles through coalescence of growing Au islands.

After adding MPEG Thiol 6000, the dispersion was centrifuged to remove small Au particles that were formed as side product in the plating step. After purification, Au nanoshell 8A1 was obtained (Fig. 5c–e and Table 1). As for Ag nanoshells, the Au nanoshells were also stable to ultrasonic treatment and centrifugation up to 6000 rpm and only segregated in time due to gravity. They were fully re-dispersible for at least 4 weeks after synthesis.

#### Structural Analysis of Nanoshell 8A1

Through FESEM analysis, we demonstrated that Au was deposited on the polystyrene particle surface and that all latex particles were covered with Au after metallization (Fig. 5b, c). The DLS measurements showed that the Au nanoshell 8A1 have a narrow size distribution and did not agglomerate, which was confirmed by FESEM analysis (Table S4). With EDS measurements, we confirmed that the shell consists, as expected, of Au (Fig. 5e). Using TEM analysis, we demonstrated that all particles were covered with Au, and no small Au particles were present in the sample after purification (Fig. 5d). TEM measurements also showed that there are still small holes in the Au shell. Nonetheless, it is visible that the seed particles grew, and the resulting islands coalesced during the electroless plating step, to form a cohesive shell. By analyzing the surface structure of the Au nanoshells using FESEM analysis, we calculated a surface coverage that was increased from <50 % for the seeded particles 7A up to 98.9 % for nanoshell 8A1. The average Au grain size was 41 nm (Table 1). It is clearly visible that, in comparison with the Ag nanoshells, the Au nanoshells display a much smoother surface structure, expressed by the small metal grain size of 41 nm (vs. 82 nm for Ag) and a higher surface coverage at comparable shell thickness (99 % for Au vs. 91 % for Ag).

#### Increase in Shell Thickness

**Fig. 6:**
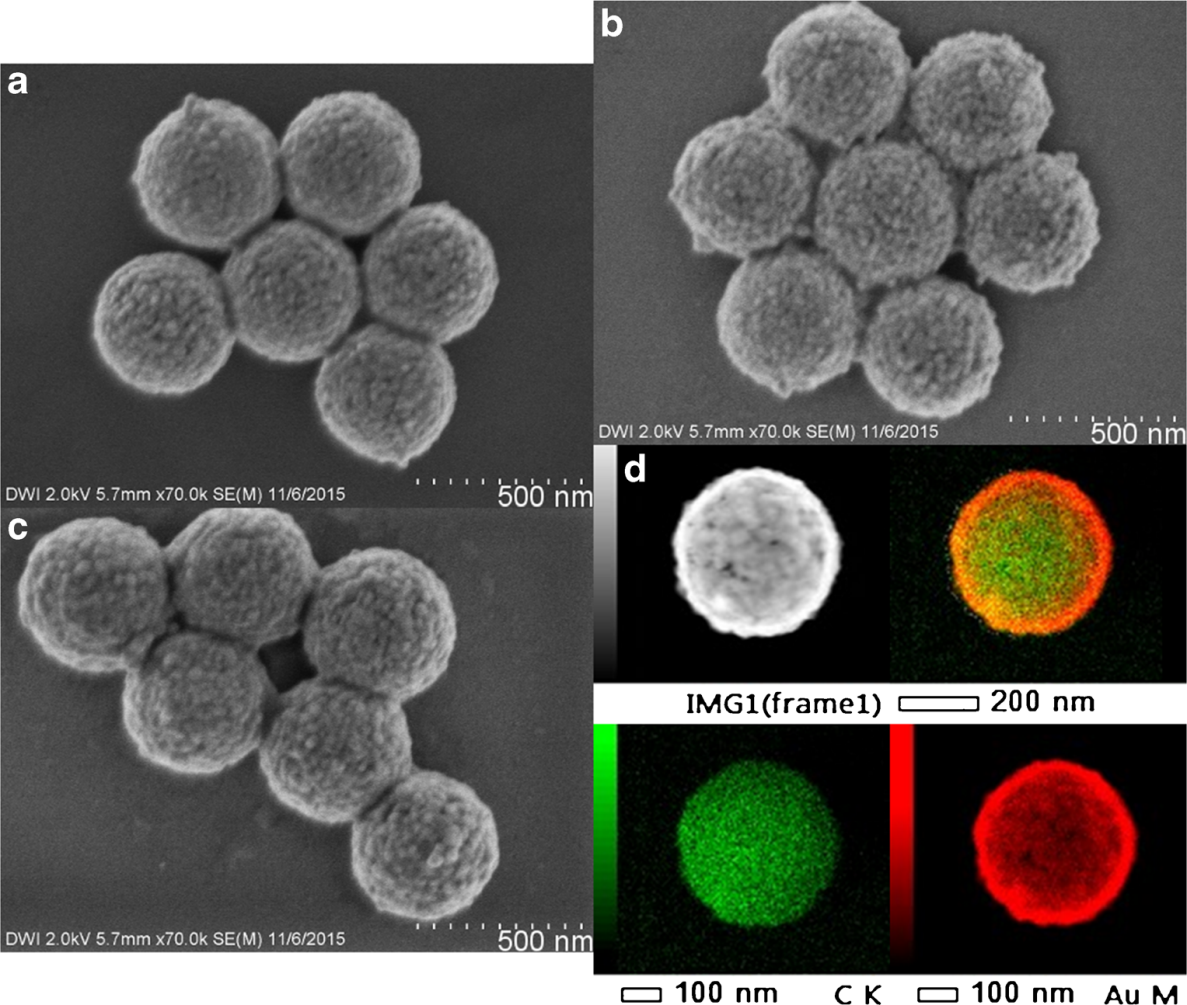
FESEM images of Au nanoshells with same core diameter and different shell thickness **a** 8A2 with 14 nm, **b** 8A3 with 20 nm, and **c** 8A4 with 24 nm shell thickness, plus **d** STEM-EDX images of nanoshells 8A4

To obtain nanoshells with an increased Au shell thickness, we changed the amount of gold(III) hydroxide solution and sodium borohydride in the plating bath. Upon doubling of the volume of gold(III) hydroxide solution and sodium borohydride, an increase of 4 nm in shell thickness was observed (Table S5). In this way, Au nanoshells 8A2–4 with shell thicknesses of 14, 20, and 24 nm were synthesized (Fig. 6 and Table 1). MPEG Thiol 6000 was added as stabilizer to avoid agglomeration (Table S4). Using FESEM, HR-TEM, STEM-HAADF, and EDS analyses, we showed that while increasing the shell thickness, the surface coverage and average grain size remained nearly constant (Table 1).

#### Decrease in Core Size

**Fig. 7:**
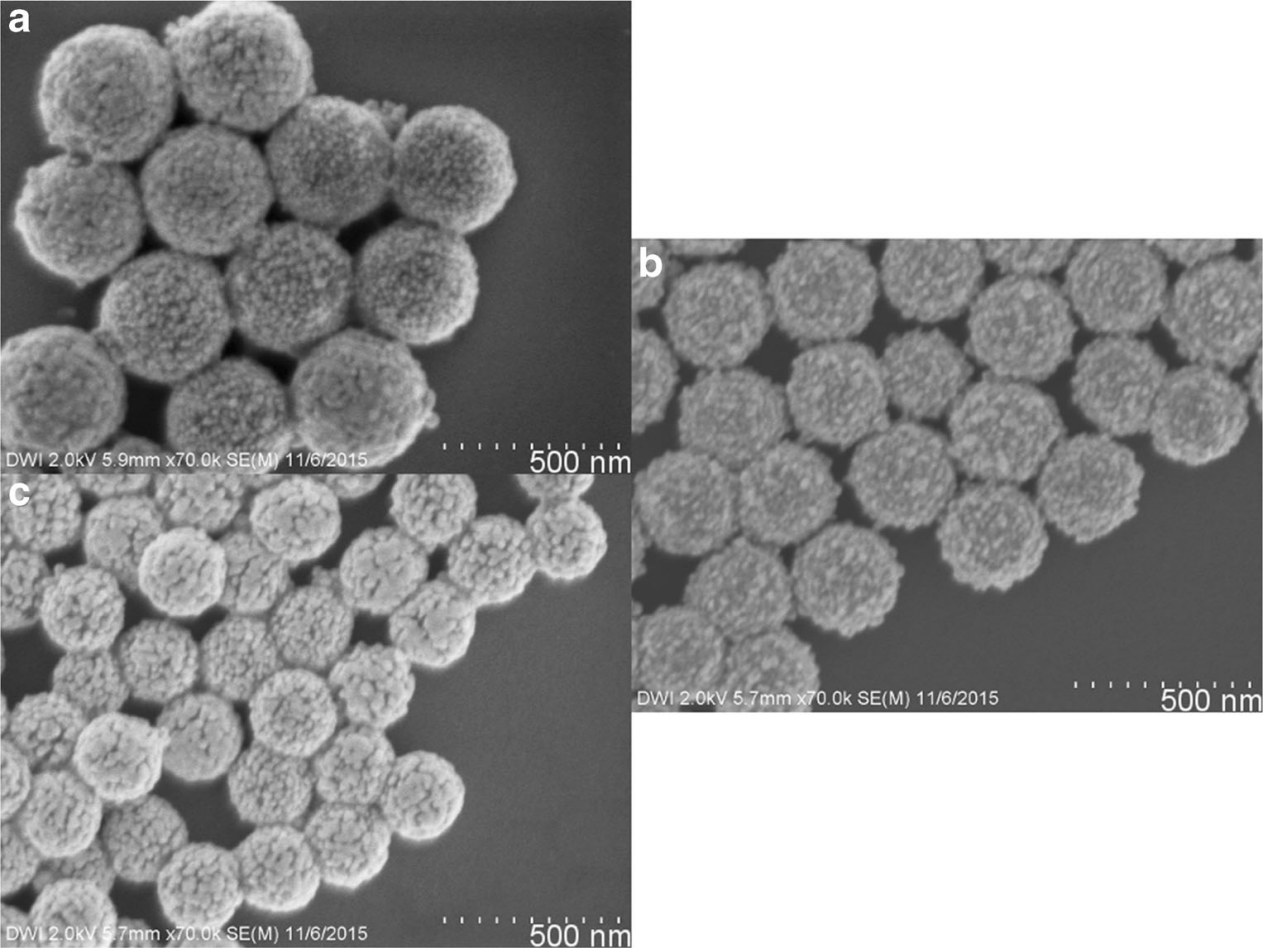
FESEM images of Au nanoshells with different core diameters and similar shell thickness **a** 8B with 339 nm, **b** 8C with 267 nm, and **c** 8D with 218 nm core diameter

By using particles with average diameter of 218 nm (4B), 267 nm (4C), and 339 nm (4D) in the above-described Au seeding and plating reactions, we synthesized Au nanoshells with different core diameters and similar shell thickness of 14–15 nm (8B–D, Fig. 7 and Table 1). To realize a similar shell thickness, the concentration of the latex particles was adjusted, so that for all experiments the same overall polystyrene particle surface area was used (Tables S1 and 5). In this way, all polystyrene particles with different core diameters were coated with a Au shell. After addition of MPEG Thiol 6000, they all showed similar stability towards centrifugation and ultrasonic treatment. Furthermore, like for all presented nanoshells, no agglomeration was observed. All Au nanoshells 8B–D display similar surface coverage and average grain size, which leads to an increased ratio of grain size to core diameter for smaller nanoshells (Table 1).

### Plasmon Resonances

The interaction of an electromagnetic field with a spherical particle can be analyzed through the calculation of the natural modes of a sphere. Stratton [47] shows that, for each electric and magnetic modes, its natural frequencies may be determined, taking into account the boundary conditions of the sphere, as the roots of specific transcendental equations which depend on the constitutive parameters of the particle and the medium where the particle is embedded in. A similar approach is made by Mie [48], in which he derived exact solutions for strong optical scattering and absorption of light by spherically symmetric particles—the so-called Mie scattering theory—an exact solution for the interaction of a plane wave with a homogeneous sphere. Therefore, the effects of absorption and scattering on such a particle—extinction cross section—may be obtained as a series of multipolar oscillations if the boundary conditions are known, and the calculations can be further simplified when the diameter of the spherical particle is much smaller than the wavelength of the radiation. Bohren and Huffmann [49] showed that, in such conditions, using the Mie’s theory, the extinction cross section of the spheres plays an important role for determining the natural frequencies of the sphere. If the imaginary parts of those complex frequencies are small when compared to the real parts, the latter are approximately the real resonance frequencies of the particle.

In 1951, Aden and Kerker [50] obtained a solution for scattering by a homogeneous sphere coated with a homogeneous layer of uniform thickness, a multi-layered sphere with radial variation of the electric and magnetic parameters of the particle. The set of equations for determining the scattering and absorption parameters increases due to the additional layer; however, the principle of determining the natural frequencies remains the same and establishes the conditions for the plasmon frequencies in metals due to their specific behavior in optical regimes.

### Optical Analysis

Previously, it has been demonstrated that metal nanoshells have highly tunable optical properties due to the strong dependence of surface resonances on the shell-to-core size ratio. It is possible to understand how nanoshells behave when excited by electromagnetic plane waves using analytical (extended Mie theory) and numerical (finite element method—FEM) approaches [47–49]. Ideal nanoshells are formed by spherical lossless dielectric nanoparticles (core) with diameter *d*_core_ and refractive index *n*_core_ covered by a concentric spherical metal layer (shell) with thickness *t*_shell_ and complex refractive index *n*_metal_ + ik_metal_. The total nanoshell diameter is given by *d*_core_ + 2*t*_shell_. In this paper, we assume that the refractive index of polystyrene is constant (*n*_ps_ = 1.59) for the whole wavelength regime of interest (300 to 1100 nm). Two different metals are studied—Au and Ag—and their optical properties are used as presented by Johnson and Christy [51].

For ideal nanoshells, extended Mie theory can be applied to fully characterize the scattering and absorption of the particles. The energy flux balance between the incident and transmitted light allows determining the rates at which the incoming energy is absorbed or scattered [52]. Following that approach, the extinction cross section (ECS) is obtained, which is the sum of the absorption and scattering cross sections.

In practical terms, in order to obtain the extinguished light, the transmitted energy flux density of the incident light (*I*_out_) is measured as a function of wavelength and compared to a reference (*I*_in_), the transmitted light through the pure solvent [49]. Therefore, the absorbance of a measured sample can be defined by Eq. (1):

1 $$ A=- \ln \left(\frac{I_{\mathrm{out}}}{I_{\mathrm{in}}}\right) $$

Absorbance is a quantity that depends on the concentration of the nanoparticles (*N*) in solution and on the path length of the light (*d*). This is modeled by the Beer-Lambert law, which describes the attenuation (*α*_ext_) of the intensity of radiation at the passage through a medium with an absorbing substance, depending on the concentration of the absorbing substance, and its thickness. The attenuation coefficient is expressed by Eq. (2).

2 $$ {\alpha}_{\mathrm{ext}}=-\frac{1}{d} \ln \left(\frac{I_{\mathrm{out}}}{I_{\mathrm{in}}}\right)=\frac{1}{d}A $$

Assuming that multiple scattering is negligible due to low concentration of particles, the ECS of a single nanoshell can be expressed in terms of absorbance of the sample with Equation (3) [49].

3 $$ \mathrm{E}\mathrm{C}\mathrm{S}=\frac{\alpha_{\mathrm{ext}}}{N}=\frac{1}{Nd}A $$

The ECS is linearly proportional to the measured absorbance (*A*), and a direct comparison between ECS and *A* is sensible to understand the plasmon resonance of metal nanoshells.

### Au Nanoshells

**Fig. 8:**
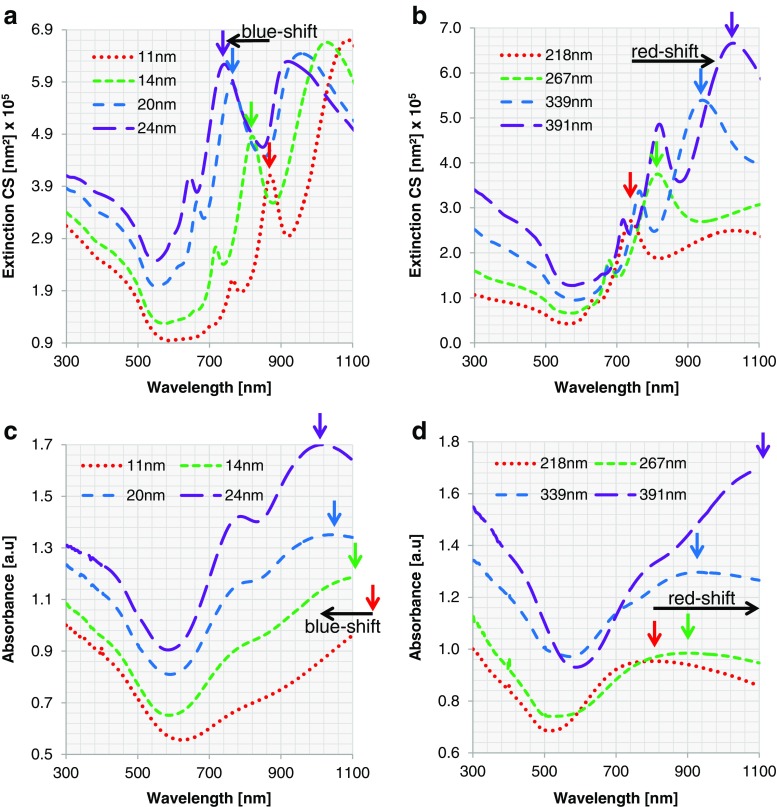
ECS for Au nanoshells with **a** different shell thicknesses and the same core diameter (391 nm) and **b** different core diameters and similar shell thickness (14 nm). Experimental measurements of absorbance of Au nanoshells for **c** different shell thicknesses with a fixed core diameter of 391 nm (8A1–4) and **d** varying core diameters with similar shell thickness of about 14 nm (8A2, 8B–D)

The plasmon resonance of Au nanoshells with varying core diameter and shell thickness is studied. For the extended Mie calculations, we assume perfect concentric spheres with a complete metal shell (100 % surface coverage) with no surface roughness. First, a fixed core of 391 nm in diameter is considered. Au nanoshells are simulated with shell thicknesses of 11, 14, 20, and 24 nm (corresponding to the samples 8A1–4). For a fixed core diameter of 391 nm, increasing shell thickness from 11 to 24 nm leads to a blueshift in extinction. This effect is displayed in both the experimentally obtained and simulated spectra (subpanels a and c of Fig. 8, respectively). However, the wavelengths of the experimentally obtained extinction maxima differ from those of the simulated ones. When we take the local maximum around 1000 nm as an example for the shift of the whole spectrum, we observe that increasing the shell thickness from 20 to 24 nm results in an experimental blueshift from 1047 to 1020 nm vs. a simulated one from 962 to 925 nm (Table S6). The experimentally obtained blueshift of this local maximum is slightly smaller than the simulated one (27 vs. 37 nm). In addition, the peaks in the experimental spectra are significantly broader than in the simulated ones, and the small local maxima present in the simulated spectra are either less pronounced or absent. Furthermore, the experimentally obtained spectra for all four particles are redshifted compared to their simulated counterparts. One reason for the redshift of about 90 nm may be the local increase in refractive index due to surface functionalization of the Au nanoshells with PEG Thiol 6000, which has a substantially larger refractive index than water (1.47 vs. 1.33). Unfortunately, this cannot be implemented into the calculations due to the unknown density of PEG Thiol 6000 and therefore the unknown exact refractive index. A second reason may be the underestimated particle size obtained from electron microscopy studies, which is likely to be larger in dispersion than in dry state.

In addition to the effect of changes in shell thickness, we studied the optical effects of changing the core diameter. For this purpose, the core diameter was varied from 218 to 267, 339, and 391 nm with a fixed Au shell of about 14 nm. Both in the experimental and the simulated spectra, a redshift is observed upon increase in core diameter from 218 to 391 nm (subpanels b and d of Fig. 8, respectively). An increase in core diameter from 218 to 267 nm, for example, causes a redshift of the local maximum from 805 to 904 nm for the experimental and from 744 to 819 nm for the simulated spectrum (Table S6). Please note that a redshift is observed in the experimental vs. simulated spectra for all nanoshells, for the same reasons as discussed above.

**Fig. 9:**
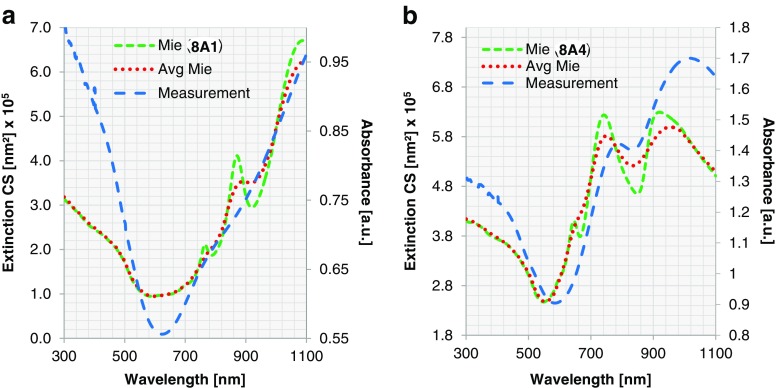
For Au nanoshells, ECS calculated for a single particle by extended Mie theory (*dashed green*; corresponding to left vertical axis) and considering a size distribution (*dotted red*; corresponding to left vertical axis) are compared with experimental measurement (*sparse dashed blue*; corresponding to right vertical axis). Two samples are shown here: **a** nanoshell 8A1 (*d*_core_ = 391 nm, *t*_shell_ = 11 nm) and **b** nanoshell 8A4 (*d*_core_ = 391 nm, *t*_shell_ = 24 nm)

For all spectra discussed until now, the peaks in the experimental spectra are significantly broader than those in the simulated ones, and the small local maxima and minima present in the simulated spectra are either less pronounced or absent. This is probably due to the size distribution of the nanoshells in the experimental samples (Table S4) [51]. To study the effect of this non-uniformity in size, we investigate further for two samples (8A1, 8A4) the effects of the variation of the core diameter following a Gaussian distribution: 21 equidistant sampling points are taken within the measured size distribution (Table S4), and extended Mie calculations are performed. Afterwards, the total ECS is calculated as a weighted sum over the Gaussian distribution for the averaging effect. Together with the measurement and the extended Mie calculation for the single particle, the calculation after averaging is shown in Fig. 9.

The extinction considering the Gaussian distribution of particle size shows broader resonance peaks, and the resonances of smaller amplitude have been smeared out. Additionally, a small redshift is observed when a size distribution is considered, which may contribute to the noted redshift of experimental vs. simulated spectra.

Evidently, the weighted curve fits better to the measurements than that of one single particle. Another observation is that the extended Mie theory describes the nanoshells better for the thicker Au shells. A possible reason for this is that for a very thin metal layer, the refractive index deviates substantially from that of the bulk material as is used in the simulations. However, this is out of the scope of this paper and will be investigated in our future work. Since all Au nanoshells fit reasonably to the calculated ECS using extended Mie theory, it can be concluded that the rather smooth surface structure and high surface coverage of Au nanoshells do not lead to any limitations when comparing the plasmonic resonances with an optimal model. We can furthermore claim that the optics of a Au nanoshell are already realised at a Au shell thickness of about 10 nm.

### Ag Nanoshells

**Fig. 10:**
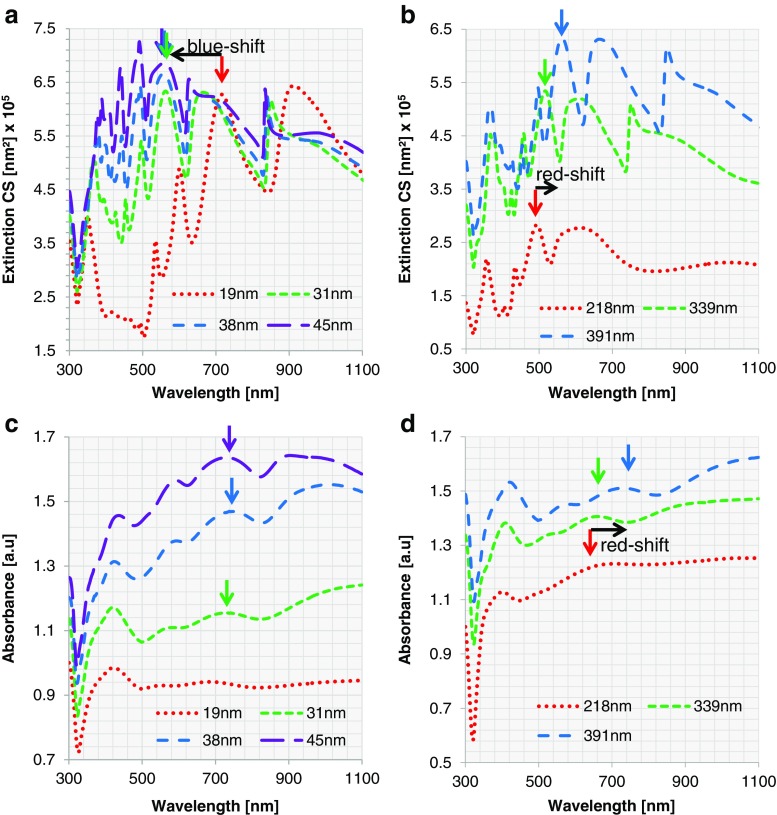
ECS for Ag nanoshells with **a** different shell thicknesses and the same core diameter (391 nm) and **b** different core diameters and similar shell thickness (around 30 nm). Experimental measurement of absorbance of Ag nanoshells: **c** for different shell thicknesses with a fixed core diameter of 391 nm (6A1–4) and **d** for varying core diameters with similar shell thicknesses around 30 nm (6A2, 6B, C)

Similar to the preceding section, the analysis is carried out for Ag nanoshells. First, for a fixed core diameter of 391 nm, ideal concentric particles are studied with Mie theory for Ag shell thicknesses of 19, 31, 38, and 45 nm (corresponding to the samples 6A1–4). By increasing the shell thickness from 19 to 31 nm, a rather large blueshift in ECS is detected (Fig. 10a). In the simulation, the local maximum at 721 nm of nanoshells 6A1 is shifted to 567 nm for nanoshell 6A2 (Table S7). By increasing the shell thickness further, the peak position remains constant and only the intensity of the ECS changes. This trend for nanoshells 6A2–4 can also be observed in the experimental data (Fig. 10c), where a local maximum can be found at a wavelength of about 740 nm for all three shell thicknesses (Table S7). The same redshift from experimentally obtained spectra in comparison to their simulated counterparts, which was observed and commented for Au nanoshells (vide supra), can also be seen here for the same reasons as described above. Nanoshell 6A1 does not fit into the overall trend. The experimentally obtained extinction curve is rather flat and the blueshift from 19 nm shell thickness to 31 nm, as observed in the simulations, cannot be observed in the experimental spectra.

The same simulation using extended Mie theory is performed for nanoshells 6B and 6D with a shell thickness of 35 and 30 nm and core diameters of 218 and 339 nm. Since in this region the change in shell thickness does not lead to a large shift in ECS, the thicknesses can be viewed as sufficiently similar for studying changes in plasmonic resonances based on changing core diameter. In the simulated spectra, a redshift can be observed with increasing core diameter (Fig. 10b).

As an example for the shift of the whole spectrum, the local maximum around 500 nm can be studied. The local maximum at 498 nm for nanoshell 6C is shifted to 521 and 567 nm for nanoshells 6B and 6A2, respectively (Table S7). The same redshift can be observed in the experimental data (Fig. 10d). Here, the local maximum at 643 nm for nanoshell 6C is shifted to 666 nm for nanoshell 6D and 731 nm for nanoshell 6A2 (Table S4). When comparing experimental to simulated spectra, again a redshift can be observed, for the same reasons as discussed in the previous section.

Although the general trend of theory and experiment matches, the shape of the curves and the positions of the local maxima differ. A few common aspects are observed from the plotted curves. For all the curves in Fig. 10, a clear dip occurs at wavelength 320 nm. This is due to the intrinsic change in the refractive index of Ag at that wavelength [51]. For all experimental measurements, a primary broad peak at about 420 nm is shown, which is absent in the theoretical model. This peak is at the same wavelength as the extinction peak of small Ag particles (Fig. S3). Since this peak does not fully disappear after multiple centrifugation cycles and after confirming via electron microscopy that no small Ag particles are present, it must be a characteristic of the surface structure. Due to the fact that there are always islands on the particle surface that did not coalesce with other islands, this plasmonic resonance of small Ag particles is still present for all Ag nanoshells. With an increase in shell thickness and surface coverage, the number of those islands is reduced and also the resonance at 420 nm becomes less prominent. All nanoshells also show a slight bump in their extinction spectra at about 360 nm, which corresponds to the local maximum at this wavelength in the calculations. In the experimental data, it is partly masked by the Ag resonance peak at 420 nm.

For a constant core diameter, the experimental measurements show a very slow increase in absorbance at wavelengths larger than 500 nm with shallow peaks at the same position. Only for the very thin layer (19 nm) the absorbance is continuously flat. The corresponding simulation, on the contrary, shows a large increase of the ECS with wavelength for the 19-nm shell thickness and a flat ECS or even a decrease in ECS for the other cases.

**Fig. 11:**
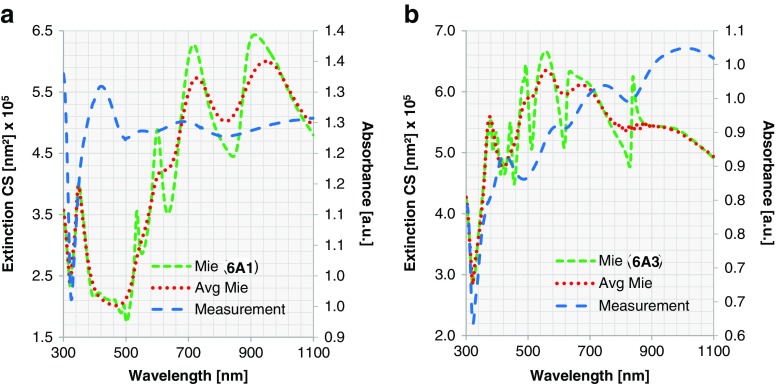
ECS calculated for a single particle by extended Mie theory (*dashed green*; corresponding to left vertical axis) and ECS considering a size distribution (*dotted red*; corresponding to left vertical axis) are compared with experimental measurement (*sparse dashed blue*; corresponding to right vertical axis). Two samples are shown here: **a** nanoshell 6A1 (*d*_core_ = 391 nm, *t*_shell_ = 19 nm) and **b** nanoshell 6A3 (*d*_core_ = 391 nm, *t*_shell_ = 38 nm)

We also observe abrupt changes around 610 and 830 nm for the other thicknesses with asymmetric Lorentz line shape. These two oscillations are determined by the core diameter. Therefore, with the fixed core size, the resonances remain at the same positions despite the change of the shell thickness. When the core diameter increases, we expect a redshift as shown in Fig. 10. When considering averaging effect of size distribution, collectively they reduce to local minima, as depicted in Fig. 11. Comparing extended Mie theory calculations for a single particle with those considering a size distribution, we see similar changes in ECS as described for Au nanoshells. The local maxima become broader and are slightly redshifted, which is in good agreement with experimental data. The ECS considering a size distribution obviously matches the experiment better than the ECS of one particle size. Nevertheless, the difference in shape of the extinction curves is still significant. This leads us to the conclusion that for Ag nanoshells, the rough surface structure and low surface coverage, especially for thin shells, result in noticeable deviations from the optimal model. This results in large constraints, when the plasmonic resonance for Ag nanoshells needs to be predicted.

When comparing Au and Ag nanoshells, it is visible that the absorbance spectra of Au nanoshells fit much better with the calculated ECS than those of Ag nanoshells. This can be explained by the higher degree of surface coverage and less pronounced roughness for Au nanoshells when compared to their Ag counterparts. Ergo, the experimentally obtained Au nanoshells resemble the perfect case of a dielectric sphere with a smooth concentric metal shell better than Ag nanoshells.

**Fig. 12:**
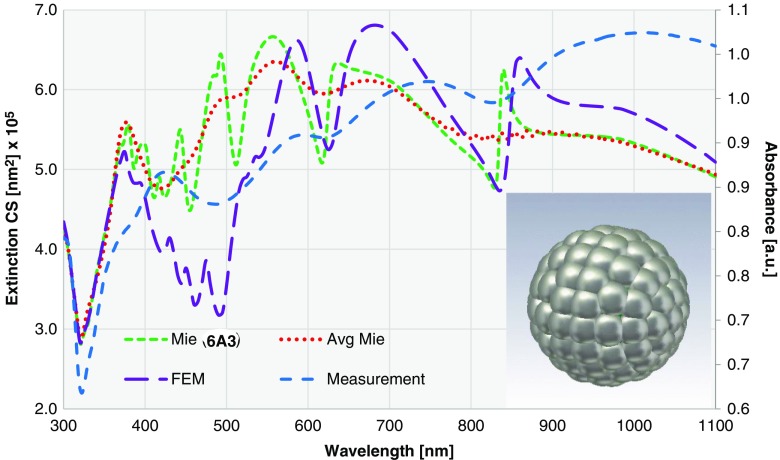
FEM simulation of the ECS for Ag nanoshell 6A3 with a core diameter of 391 nm and average Ag shell thickness of 38 nm (*long dashed purple*; corresponding to left vertical axis) and comparison with the measurement (*sparse dashed blue*; corresponding to right vertical axis), the extended Mie theory (*dashed green*; corresponding to left vertical axis) and the averaged extended Mie theory (*dotted red*; corresponding to left vertical axis) plus Ag nanoshell geometry for FEM simulations (*inlay*)

As described in the previous sections, when Ag shells are synthesized on the core material by seeded growth, small Ag seeds grow into islands, which ultimately coalesce to form a rough, cauliflower-type shell. As the extended Mie model is built using smooth concentric spherical geometry, it does not give absolute satisfactory explanation for the granular Ag nanoshells. To study the influence of the grains on the surface of the nanoshells, a numerical simulation is performed using finite element method (CST Studio Suite®; Fig. 12).

FEM simulation takes into account the surface coverage and average grain size (Table 1). The geometry of the simulated particle is illustrated in Fig. 12. With the granular surface, the ECS peaks redshift and the ECS peaks between 400 and 500 nm vanish. Above 500 nm, there are three distinct maxima visible that correspond well to the experimental data. The experimentally obtained extinction peaks are still redshifted in comparison with FEM calculations, but the shape of both curves matches much better. This indicates that the topography of the Ag shell has a clear influence on the optical properties. More systematic study including granularity, surface coverage, size distribution, etc. will be the subject of a follow-up paper.

## Conclusion

Both for Au and Ag nanoshells, we successfully studied the effect of non-uniformity in core size on their plasmon resonance and unveiled the metal shell thickness required to realize the plasmon resonance of a metal nanoshell. We successfully prepared Au and Ag nanoshells through selective deposition of the metal on glucose-functionalized polystyrene particles in a seed growth process. We produced polystyrene particles with diameters ranging from 218 to 391 nm and Au and Ag shells of a thickness ranging from 11 to 24 nm and 19 to 45 nm, respectively. Both the polystyrene particles and the Ag and Au nanoshells displayed a size distribution of about 5 %. Using electron microscopy, we determined that both the Ag and Au nanoshells were incomplete and rough: the degree of surface coverage of the polystyrene particles was about 98 % for a Au shell thickness from 11 nm onwards, while the surface coverage for a 19-nm-thick Ag shell was only 91 %. The metal grains forming the nanoshell were about twice as large for Ag than for Au. To realize a Ag surface coverage of 98 %, a 45-nm-thick shell was required. For Ag nanoshells with small cores (218 nm), the shell roughness led to large deviations from ideal concentric nanoshells.

We studied the plasmon resonance of aqueous dispersions of Ag and Au nanoshells using extinction spectrophotometry and compared the results to simulated spectra obtained using the extended Mie theory. For Au nanoshells, we observed a reasonable match between all experimental spectra and their simulated counterparts, albeit that the peak width in the experimental spectra was significantly broader, causing local maxima to smear out or even disappear. This discrepancy could largely be attributed to the particle size distribution, as confirmed through the extended Mie simulations using Gaussian size distributions obtained from SEM analyses. Ergo, we conclude that even for very thin Au shells of 11 nm, the optics of a Au nanoshell are realized.

In case of Ag nanoshells, the match between all experimental spectra and their simulated counterparts was significantly worse. Especially for thin Ag shells of 19 nm, the discrepancy between the experimental spectra and those obtained through the extended Mie simulations was large. This could be attributed to the large shell roughness, with Ag grains twice the size of their Au counterparts, and the incomplete surface coverage. Using FEM simulations taking into account Ag grain size and surface coverage, we realized significantly better fits between experimental and simulated spectra. Based on these studies, we conclude that a minimum Ag shell thickness of 30 nm is required to realize the optics of a Ag nanoshell. Ag nanoshells are sensitive towards oxidation and require synthesis and storage under inert gas. We confirmed that oxidation strongly affects the optics of the nanoshells.

In conclusion, we have demonstrated that particle size distribution and metal shell imperfections in Ag and Au nanoshells are intrinsic to the synthesis of such particles and therefore largely unavoidable. As demonstrated, these have a strong impact on the plasmon resonance of the resulting nanoshells, which may limit their potential for application, and at least needs to be taken into account when designing such shells for specific applications. Currently, we are investigating the potential of Au and Ag nanoshells as carrier for catalytically active materials in plasmon catalysis.

### Experimental Section

#### Materials

α-d-(+)-Glucose (anhydrous, 96 %), silver nitrate (>99 %), chloroauric acid (≥99.9 %), sodium borohydride (99 %), and poly(ethylene glycol) methyl ether thiol (average *M*_n_ 6000) were purchased from Sigma-Aldrich. Tin(II) chloride (anhydrous) and aqueous ammonia (25 wt %) and were purchased from Merck. Ethanol (absolute) was purchased from VWR BDH Prolabo Chemicals. l-(+)-Tartaric acid (99 %) was purchased from Alfa Aesar. Hydrochloric acid was purchased from TH Geyer. Sodium hydroxide (≥99 %) was purchased from Carl Roth. Formaldehyde was purchased from KFM optiChem. Potassium carbonate was purchased from Fluka. Glucose-functionalized polystyrene particles were synthesized according to a previously published procedure (Table S1) [44].

#### Silver Seeding

Latex dispersion 4A (150 μL, 10.9 mg/mL, 1.46 × 10^16^ nm^2^/mg) was added to a solution of tin(II) chloride (42 mg) and hydrochloric acid (100 μL, 37 %) in water (20 mL). The mixture was stirred for 1 h at room temperature and afterwards centrifuged three times for 30 min at 6000 rpm and dispersed again in degassed water (20 mL) using an ultrasonic bath. Subsequently, the mixture was added under N_2_ atmosphere to a solution of silver nitrate (5 mg) and aqueous ammonia (75 μL, 25 wt%) in degassed water (20 mL). An immediate color change showed the reduction of silver ions. MPEG Thiol 6000 (10 mg) in 2 mL degassed water was added, and the reaction mixture was centrifuged for 30 min at 6000 rpm and redispersed in 10 mL degassed water.

Silver seeding for latex dispersions 4B and 4D was performed according to above procedure. Volume of latex dispersions used was calculated to keep a constant overall particle surface (Table S3).

#### Silver Plating

A solution of glucose (60 mg), tartaric acid (6 mg), and ethanol (150 μL) in degassed water (30 mL) under N_2_ atmosphere was heated under reflux for 2 h. After cooling to room temperature, 7 mL of this solution was added, under N_2_ atmosphere, to a solution of silver nitrate (7 mg), aqueous ammonia (100 μL, 25 wt%), and sodium hydroxide (10 mg) in 100 mL degassed water in an ice bath. The polystyrene silver composite particle dispersion 5A (10 mL, 0.16 mg/mL) was added, and the reaction mixture was stirred for 1 h at 0 °C. MPEG Thiol 6000 (10 mg) in 2 mL degassed water was added, and the reaction mixture was centrifuged for 30 min at 2000 rpm and dispersed again in degassed water.

Synthesis of polystyrene silver core shell particles 6A2–4 and 6B, C was performed according to above procedure by adjusting the amount of silver diammine and glucose solution or by using composite particles 5B, C (Table S3).

#### Gold Seeding

Latex dispersion 4A (250 μL, 10.9 mg/mL, 1.46 × 10^16^ nm^2^/mg) was added to a solution of tin(II) chloride (42 mg) and hydrochloric acid (100 μL, 37 %) in water (20 mL). The mixture was stirred for 1 h at room temperature and afterwards centrifuged three times for 30 min at 6000 rpm and dispersed again in demineralized water (20 mL) using an ultrasonic bath. Subsequently, the mixture was added to a solution of chloroauric acid (5 mg) and potassium carbonate (5 mg) in water (20 mL). An aqueous formaldehyde solution (100 mL, 2 wt%) was added, and the reaction mixture was stirred for 1 h at 80 °C. After cooling to room temperature, the mixture was centrifuged for 30 min at 6000 rpm and dispersed again in 10 mL demineralized water.

Gold seeding for latex dispersions 4B–D was performed according to above procedure. Volume of latex dispersions used was calculated to keep a constant overall particle surface (Table S5).

#### Gold Plating

Chloroauric acid (45 mg) and potassium carbonate (180 mg) were dissolved in water (300 mL) and aged overnight to create a gold hydroxide solution. The polystyrene gold composite particle dispersion 7A (10 mL, 0.27 mg/mL) was combined with 75 mL of the gold hydroxide solution. A solution of sodium borohydride (2 mg) in 7.5 mL water was added with a syringe pump in 1 h under stirring at room temperature. MPEG Thiol 6000 (10 mg) in 2 mL water was added, and the reaction mixture was centrifuged for 30 min at 2000 rpm and dispersed again in demineralized water.

Synthesis of polystyrene gold core shell particles 8A2–4 and 8B–D was performed according to above procedure by adjusting the amount of gold hydroxide and sodium borohydride solution or by using composite particles 7B–D (Table S5).

#### Instruments and Methods

Extended Mie theory was implemented as a MATLAB® code based on the formulations presented by Bohren and Huffman [48] and Kerker and Loebl [53].

Numerical simulations were performed in frequency domain using CST Studio Suite® 2015 SP4 (CST MWS) using 12 steps of adaptive tetrahedral mesh refinement. The nanoparticles were excited by an electromagnetic plane wave polarized in *x*-direction. Boundary conditions were defined as “open (add space)” and the background, water (*n*_water_ = 1.33). For perfect spherical nanoparticles, perfect electric conductor at *yz*-plane and perfect magnetic conductor at *xz*-plane were used as symmetry conditions. The method is first benchmarked with the extended Mie theory for ideal nanoshells and then is implemented for the complex granular nanoshell. The granular nanoshell is modeled as a spherical dielectric core surrounded by metallic oblate ellipsoids. The two major axes are equal to the grain size and orthogonal to the core radius. The minor axis is equal to the shell thickness. The final total number of mesh cells (tetrahedrons) is 222.579, in which the length of the shortest mesh edge is 0.0418514 nm, and the longest mesh edge is 62.1708 nm.

UV/Vis measurements were conducted at 25 °C using a Jasco V-6300 UV/Vis spectrometer with a range from 190 to 1100 nm. Therefore, disposable polystyrene cuvettes from Brand GMBH & CO KG were used.

FESEM images were acquired using a Hitachi S4800 FESEM. For sample preparation, one droplet of the particle dispersion was placed on a silicon wafer and dried at room temperature. The samples were sputtered with gold. Particle diameters from FESEM images were determined using MATLAB. For average diameter and size distribution, at least 200 particles per sample were measured.

TEM studies were performed using a JEOL ARM 200 probe-corrected TEM, operated at 200 kV. Imaging of the particles was performed in high-angle annular dark field (HAADF)-scanning TEM (STEM) mode. EDS spectra were recorded using a 100-mm^2^ Centurio SDD detector. EDS mappings were obtained in STEM mode by acquiring full spectra in grids of either 256 × 256 or 512 × 512 pixels. All mappings were obtained by summation of 50–100 frames, each having 0.1 ms acquisition time per pixel per frame. In this way, the particles remained unaffected by the impact of the incident electron beam.

XPS measurements were carried out in an Ultra Axis^TM^ spectrometer (Kratos Analytical, Manchester, UK). The samples were irradiated with monoenergetic Al K_α1,2_ radiation (1486.6 eV), and the spectra were taken at a power of 144 W (12 kV × 12 mA). The aliphatic carbon (C–C, C–H) at a binding energy of 285 eV (C 1s photoline) was used to determine the charging.

DLS measurements were performed at 25 °C using a Zetasizer Nano Series (Malvern Instruments). Disposable polystyrene cuvettes (Brand) were used, and for one measurement, 10–15 cycles with a measuring time of 10 s were averaged. The average figure of three separate measurements was calculated.

## Electronic Supplementary Material

Below is the link to the electronic supplementary material.

ESM 1 Additional reaction schemes, particle characteristics, XPS data, reaction parameters, and UV/Vis data. (DOCX 7246 kb)
