# Supplementary material for: The Influence of Particle Size Distribution and Shell Imperfections on the Plasmon Resonance of Au and Ag Nanoshells
Source: Plasmonics. 2016 Aug 8;12(3):929–45. doi: 10.1007/s11468-016-0345-8 (PMC5420003; doi:10.1007/s11468-016-0345-8)
Supplement: Supplementary file 1 — Additional reaction schemes, particle characteristics, XPS data, reaction parameters, and UV/Vis data. (DOCX 7246 kb) [file 11468_2016_345_MOESM1_ESM.docx]

Supporting Information

The Influence of Particle Size Distribution and Shell Imperfections on the Plasmon Resonance of Au and Ag Nanoshells

*Daniel Mann, ^†^ Daniel Nascimento-Duplat, ^‡^ Helmut Keul, ^†^ Martin Möller, ^†^ Marcel Verheijen,^§,∥^ Man Xu, ^‡,⊥^ Paul H. Urbach, ^‡^ Aurèle J.L. Adam,^*,‡^ Pascal Buskens^†,⊥^.*

^†^ DWI – Leibniz Institute for Interactive Materials e.V., Forckenbeckstr. 50, 52056 Aachen, Germany.

^‡^ Delft University of Technology, Department of Imaging Physics, Lorentzweg 1, 2628 CJ, Delft, The Netherlands.

^§^ Philips Innovation Labs, High Tech Campus 11, 5656 AE, Eindhoven, The Netherlands.

^∥^ Eindhoven University of Technology, Department of Applied Physics, P. O. Box 513, 5600 MB, Eindhoven, The Netherlands.

^⊥^ The Netherlands Organisation for Applied Scientific Research (TNO), De Rondom 1, 5612 AP, Eindhoven, The Netherlands.

Corresponding Author:

A. J. L. ADAM, [a.j.l.adam@tudelft.nl](mailto:a.j.l.adam@tudelft.nl), phone: + 31.15.2782455, Fax + 31.15.2788105

**Scheme S1.** Synthesis of glucose functionalized polystyrene particles (D. Mann, S. Chattopadhyay, S. Pargen, M. Verheijen, H. Keul, P. Buskens, M. Möller, *RSC Adv.*, 2014, **4**, 62878).

**Table S1.** Characteristics of polystyrene template particles **4A-D**.

| particle | diameter^a)^ [nm] | distribution^b)^ | PDI (DLS) | concentration [mg/mL] | particle surface area [nm^2^/mg] |
| --- | --- | --- | --- | --- | --- |
| 4A | 391 | 0.036 | 0.024 | 10.9 | 1.46 x10^16^ |
| 4B | 339 | 0.032 | 0.034 | 7.1 | 1.69 x10^16^ |
| 4C | 267 | 0.041 | 0.019 | 1.7 | 2.14 x10^16^ |
| 4D | 218 | 0.050 | 0.042 | 2.1 | 2.62 x10^16^ |

a) Average size calculated from FESEM images using at least 200 particles.

b) Distribution calculated from FESEM images by dividing the standard deviation by average size of at least 200 particles.

**Table S2.** Particle dimensions of Ag nanoshells stabilized with MPEG Thiol 6000.

| particle | diameter^a)^ [nm] | distribution^b)^ | diameter (DLS) [nm] | PDI^c)^ (DLS) |
| --- | --- | --- | --- | --- |
| **6A1** | 429 | 0.028 | 440 | 0.042 |
| **6A2** | 452 | 0.028 | 466 | 0.056 |
| **6A3** | 466 | 0.031 | 478 | 0.057 |
| **6A4** | 481 | 0.029 | 497 | 0.053 |
| **6B** | 409 | 0.031 | 418 | 0.036 |
| **6C** | 278 | 0.032 | 292 | 0.045 |

a) Average diameter calculated from FESEM images using at least 200 particles.

b) Distribution calculated from FESEM images by dividing the standard deviation by average size of at least 200 particles.

c) The polydispersity index (PDI) describes the size distribution calculated from DLS measurements. A narrow size distribution is characterized with a PDI <0.1 (International Standard ISO13321 *Methods for Determination of Particle Size Distribution Part 8: Photon Correlation Spectroscopy*, International Organisation for Standardisation (ISO) 1996; International Standard ISO22412 *Particle Size Analysis - Dynamic Light Scattering*, International Organisation for Standardisation (ISO) 2008.)


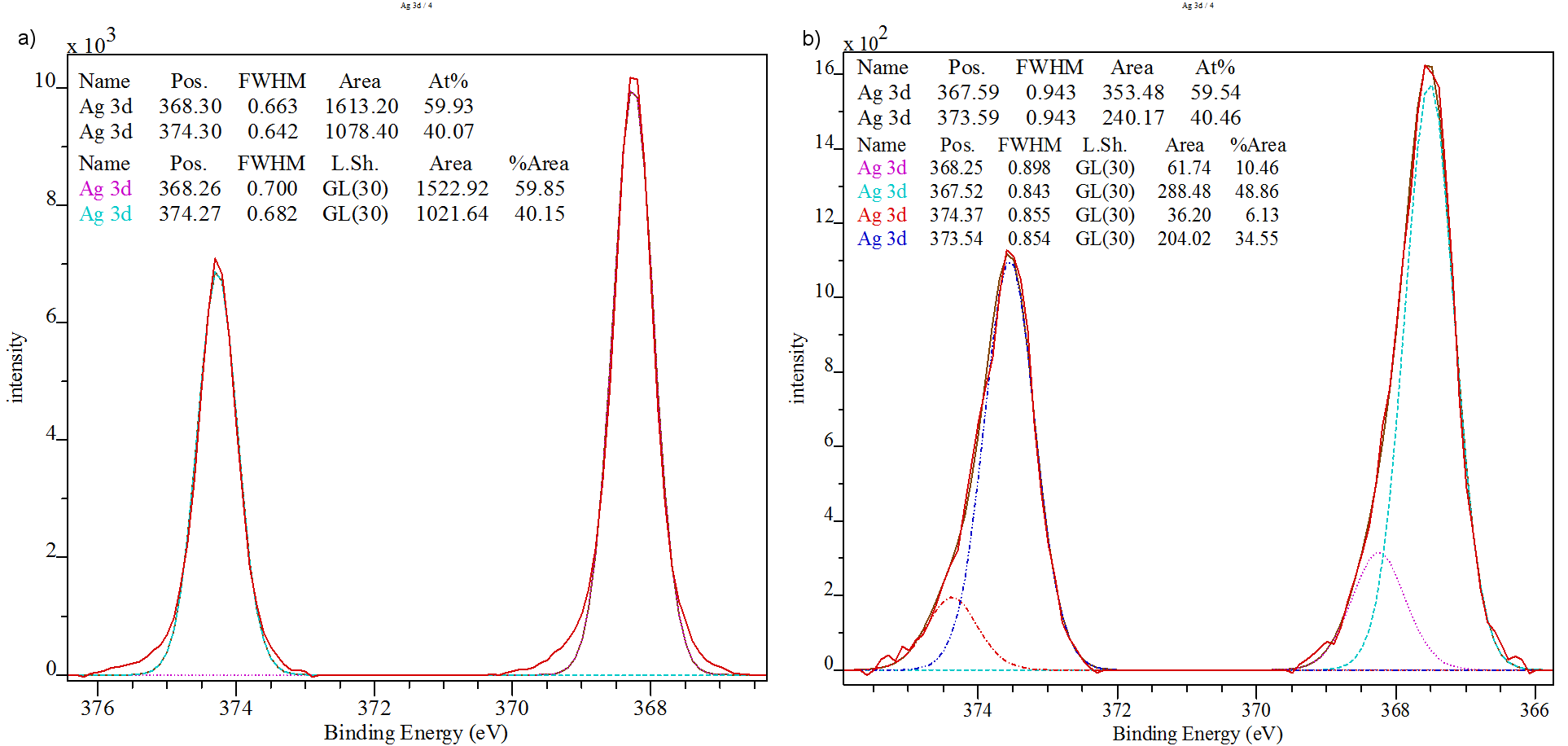


**Figure S1.** High resolution XPS data of the Ag 3d region for Ag nanoshells synthesized a) under nitrogen atmosphere and b) in air.

**Figure S2.** Optical properties of Ag nanoshells with 391 nm core diameter and 45 nm shell thickness synthesized under N_2_ atmosphere (dashed red) and in air (sparse dashed green).

**Table S3.** Silver seeding and plating to Ag nanoshells: Reagents and conditions.

| particle | seeding | | plating | | | |
| --- | --- | --- | --- | --- | --- | --- |
|  | latex dispersion | | composites | | silver nitrate [mg] | glucose solution^a)^ [mL] |
|  | particle | volume [mL] | particles | concentration [mg/mL] |  |  |
| **6A1** | **4A** | 0.15 | **5A** | 0.16 | 7 | 7 |
| **6A2** | **4A** | 0.15 | **5A** | 0.16 | 10 | 10 |
| **6A3** | **4A** | 0.15 | **5A** | 0.16 | 15 | 15 |
| **6A4** | **4A** | 0.15 | **5A** | 0.16 | 20 | 20 |
| **6B** | **4B** | 0.2 | **5B** | 0.14 | 7 | 7 |
| **6C** | **4D** | 0.4 | **5C** | 0.08 | 7 | 7 |

a) Concentration of glucose solution: c = 11.1 mol/L

All reactions were carried out in degased water under N_2_ atmosphere; for seeding particle dispersions were first stirred with 1.77 g/m^2^ particle surface tin(II) chloride and 100µL hydrochloric acid; 0.21 mg/m^2^ particle surface silver nitrate was used to synthesize Ag nanoshells; for seeding and plating ammonia solution was added to silver nitrate to form silver diamine complex; for plating sodium hydroxide was added to adjust pH; glucose solution consists of glucose, tartaric acid and ethanol in degased water.

**Table S4.** Particle dimensions of Au nanoshells stabilized with MPEG Thiol 6000.

| particle | diameter^a)^ [nm] | distribution^b)^ | diameter (DLS) [nm] | PDI^c)^ (DLS) |
| --- | --- | --- | --- | --- |
| **8A1** | 413 | 0.029 | 425 | 0.048 |
| **8A2** | 418 | 0.027 | 428 | 0.043 |
| **8A3** | 430 | 0.027 | 445 | 0.052 |
| **8A4** | 438 | 0.026 | 452 | 0.055 |
| **8B** | 367 | 0.038 | 379 | 0.041 |
| **8C** | 297 | 0.038 | 311 | 0.035 |
| **8D** | 248 | 0.029 | 257 | 0.046 |

a) Average diameter calculated from FESEM images using at least 200 particles.

b) Distribution calculated from FESEM images by dividing the standard deviation by average size of at least 200 particles.

c) The polydispersity index (PDI) describes the size distribution calculated from DLS measurements. A monodisperse (narrow) size distribution is characterized with a PDI <0.1 (International Standard ISO13321 *Methods for Determination of Particle Size Distribution Part 8: Photon Correlation Spectroscopy*, International Organisation for Standardisation (ISO) 1996; International Standard ISO22412 *Particle Size Analysis - Dynamic Light Scattering*, International Organisation for Standardisation (ISO) 2008.)

**Table S5.** Gold seeding and plating to Au nanoshells: Reagents and conditions.

| particle | seeding | | plating | | | |
| --- | --- | --- | --- | --- | --- | --- |
|  | latex dispersion | | composites | | gold hydroxide solution^a)^ [mL] | sodium boro-hydride [mg] |
|  | particle | volume [mL] | particles | concentration [mg/mL] |  |  |
| **8A1** | **4A** | 0.25 | **7A** | 0.27 | 40 | 1 |
| **8A2** | **4A** | 0.25 | **7A** | 0.27 | 75 | 2 |
| **8A3** | **4A** | 0.25 | **7A** | 0.27 | 150 | 4 |
| **8A4** | **4A** | 0.25 | **7A** | 0.27 | 300 | 8 |
| **8B** | **4B** | 0.3 | **7B** | 0.21 | 75 | 2 |
| **8C** | **4C** | 1.0 | **7C** | 0.17 | 75 | 2 |
| **8D** | **4D** | 0.7 | **7D** | 0.15 | 75 | 2 |

a) Concentration of gold hydroxide solution: c = 0.38 mol/L

All reactions were carried out in water; for seeding particle dispersions were first stirred with 1.06 g/m^2^ particle surface tin(II) chloride and 100µL hydrochloric acid; for seeding potassium carbonate was added to adjust the pH and formaldehyde was used as reducing agent; 0.25 mg/m^2^ particle surface chloroauric acid was used to synthesize Au nanoshells; gold hydroxide solution for plating was prepared fresh by stirring chloroauric acid with potassium carbonate in water over night; sodium borohydride was added with a syringe pump over one hour.

**Table S6.** Local extinction maxima of Au nanoshells, calculated by Mie theory and experimental data.

|  | | local maximum 1 | local maximum 2 | local maximum 3 |
| --- | --- | --- | --- | --- |
| **8A1** | Mie theory | 770 nm | 875 nm | 1091 nm |
|  | experimental | - | - | >1100 nm |
| **8A2** | Mie theory | 723 nm | 824 nm | 1032 nm |
|  | experimental | - | 830 nm | >1100 nm |
| **8A3** | Mie theory | 670 nm | 767 nm | 962 nm |
|  | experimental | - | 813 nm | 1047 nm |
| **8A4** | Mie theory | 650 nm | 742 nm | 925 nm |
|  | experimental | - | 792 nm | 1020 nm |
| **8B** | Mie theory | 682 nm | 768 nm | 945 nm |
|  | experimental | - | 740 nm | 932 nm |
| **8C** | Mie theory | - | 683 nm | 819 nm |
|  | experimental | - | - | 904 nm |
| **8D** | Mie theory | - | 640 nm | 744 nm |
|  | experimental | - | - | 805 nm |

**Table S7.** Local extinction maxima of Ag nanoshells, calculated by Mie theory and experimental data.

|  | | local maximum 1 | local maximum 2 | local maximum 3 | local maximum 4 |
| --- | --- | --- | --- | --- | --- |
| **6A1** | Mie theory | 354 nm | 540 nm | 605 nm | 721 nm |
|  | experimental | 426 nm | 558 nm | 689 nm | - |
| **6A2** | Mie theory | 371 nm | 458 nm | 501 nm | 567 nm |
|  | experimental | 425 nm | 589 nm | 731 nm | >1100 nm |
| **6A3** | Mie theory | 382 nm | 447 nm | 497 nm | 561 nm |
|  | experimental | 429 nm | 598 nm | 747 nm | 1018 nm |
| **6A4** | Mie theory | 383 nm | 444 nm | 496 nm | 563 nm |
|  | experimental | 441 nm | 596 nm | 739 nm | 908 nm |
| **6B** | Mie theory | 374 nm | 428 nm | 462 nm | 521 nm |
|  | experimental | 414 nm | 517 nm | 666 nm | 946 nm |
| **6C** | Mie theory | 362 nm | 409 nm | 440 nm | 498 nm |
|  | experimental | 407 nm | - | 643 nm | 1053 nm |


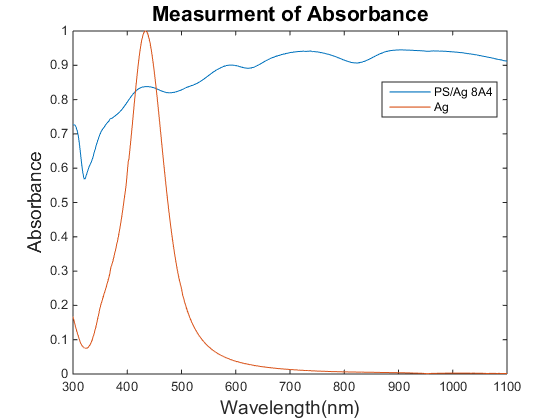


**Figure S3.** Measured absorbance by Ag nanoshells **6A4** (blue) and pure Ag nanoparticles with 50 nm average diameter (red).
